# Non-Pharmacological Interventions for Sleep Quality and Related Symptoms in Patients with End-Stage Renal Disease: A Systematic Review and Meta-Analysis

**DOI:** 10.3390/healthcare14182921

**Published:** 2026-09-09

**Authors:** Xiu Huang, Xiya Ren, Yajie Hao, Limei Zhao, Zhibo Zhao, Rongrong Wang, Xiaoshuang Zhou

**Affiliations:** 1 Department of Nephrology, The Fifth Clinical Medical College of Shanxi Medical University (Shanxi Provincial People’s Hospital), Taiyuan 030012, China; 2 Department of Nephrology, Shanxi Provincial People’s Hospital, Taiyuan 030012, China; 3 Big Data Center of Kidney Disease, Shanxi Provincial People’s Hospital, Taiyuan 030012, China

**Keywords:** end-stage renal disease, dialysis, insomnia, sleep quality, non-pharmacological interventions, systematic review, meta-analysis, randomized controlled trial

## Abstract

Background: Sleep-related problems are common among patients receiving dialysis for end-stage renal disease (ESRD), while pharmacological management may be complicated by comorbidities, polypharmacy, and altered drug clearance. This systematic review and meta-analysis evaluated the effects of non-pharmacological interventions on sleep-related outcomes, anxiety symptoms, depressive symptoms, and fatigue symptoms in this population. Methods: PubMed, Embase, the Cochrane Library, and Web of Science were searched from inception to February 2026. The prespecified eligibility criterion was restricted to randomized controlled trials (RCTs) involving adults with ESRD receiving hemodialysis or peritoneal dialysis and reporting an eligible sleep-related outcome. One quasi-cluster randomized trial was retained as a documented protocol deviation. Standardized mean differences (SMDs) calculated using Hedges’ *g* and 95% confidence intervals (CIs) were pooled using random-effects models with restricted maximum-likelihood estimation and the Hartung–Knapp–Sidik–Jonkman adjustment (REML–HKSJ). Risk of bias was assessed using the revised Cochrane Risk of Bias tool for randomized trials (RoB 2), and evidence certainty was evaluated using the Grading of Recommendations Assessment, Development and Evaluation (GRADE) approach. PROSPERO: CRD420251123449. Results: The 19 RCTs and one quasi-cluster randomized trial contributed an effective sample size of 1294 participants. The pooled estimate favored non-pharmacological interventions for sleep-related outcomes (Hedges’ *g* = −0.820, 95% CI −1.188 to −0.451; *p* = 0.0002), with substantial heterogeneity (*I*^2^ = 83.1%; *τ*^2^ = 0.4879). The 95% prediction interval (PI) ranged from −2.325 to 0.685. The pooled effects on anxiety symptoms (Hedges’ *g* = −0.29, 95% CI −0.99 to 0.41; *p* = 0.219), depressive symptoms (Hedges’ *g* = −0.64, 95% CI −2.32 to 1.04; *p* = 0.313), and fatigue symptoms (Hedges’ *g* = −0.41, 95% CI −0.93 to 0.11; *p* = 0.087) were not statistically significant. Effect estimates differed significantly by comparator type (Q-between = 11.28, df = 2; *p* = 0.0036). Conclusions: Non-pharmacological interventions may improve sleep-related outcomes in patients receiving dialysis for ESRD. However, substantial heterogeneity and a prediction interval crossing the null value limit confidence in the pooled estimate. Current evidence does not demonstrate clear benefits for anxiety symptoms, depressive symptoms, or fatigue symptoms.

## 1. Introduction

Chronic kidney disease (CKD) is defined as abnormalities of kidney structure or function, present for a minimum of 3 months, with implications for health [1]. Recent epidemiological data indicate that the global prevalence of CKD is as high as 9.1% [2]. The number of patients with CKD progressing to ESRD is expected to continue to increase annually [3]. Kidney replacement therapy for ESRD primarily comprises dialysis and kidney transplantation. The growing demand for these treatments and the associated substantial health-care and economic burden have made CKD a major global public health challenge.

Sleep-related problems, including insomnia symptoms and poor subjective sleep quality, are common among patients receiving dialysis. Restless legs syndrome (RLS) is a distinct sensorimotor disorder but may also substantially disrupt sleep in this population. Poor sleep among patients receiving dialysis has been associated with daytime dysfunction, impaired health-related quality of life, fatigue, depression, anxiety, increased health-care utilization, and a higher risk of mortality [3,4], thereby contributing to a substantial individual and societal burden. Management of sleep-related problems may involve both pharmacological and non-pharmacological interventions. However, patients receiving dialysis frequently have multiple comorbidities, polypharmacy, and altered drug clearance, while sedative–hypnotic medications may cause cognitive and psychomotor impairment and increase the risk of falls [5]. The use of these medications therefore requires particular caution in this population. These concerns further underscore the need to identify safe and effective non-pharmacological strategies for managing sleep-related problems in patients receiving dialysis.

A previous systematic review suggested that non-pharmacological interventions, including cognitive behavioral therapy for insomnia (CBT-I), exercise training, and acupressure, may improve sleep quality in patients with dialysis-dependent ESRD [6]. However, the available evidence was limited by the small number of studies and heterogeneity in participant eligibility criteria and comparator conditions. We therefore conducted an updated systematic review and meta-analysis focused on randomized evidence to evaluate the effects of non-pharmacological interventions on sleep-related outcomes, anxiety symptoms, depressive symptoms, and fatigue symptoms. Anxiety and depressive symptoms were not assessed in the previous review.

## 2. Materials and Methods

### 2.1. Reporting Guidelines and Protocol Registration

**Figure 1:**
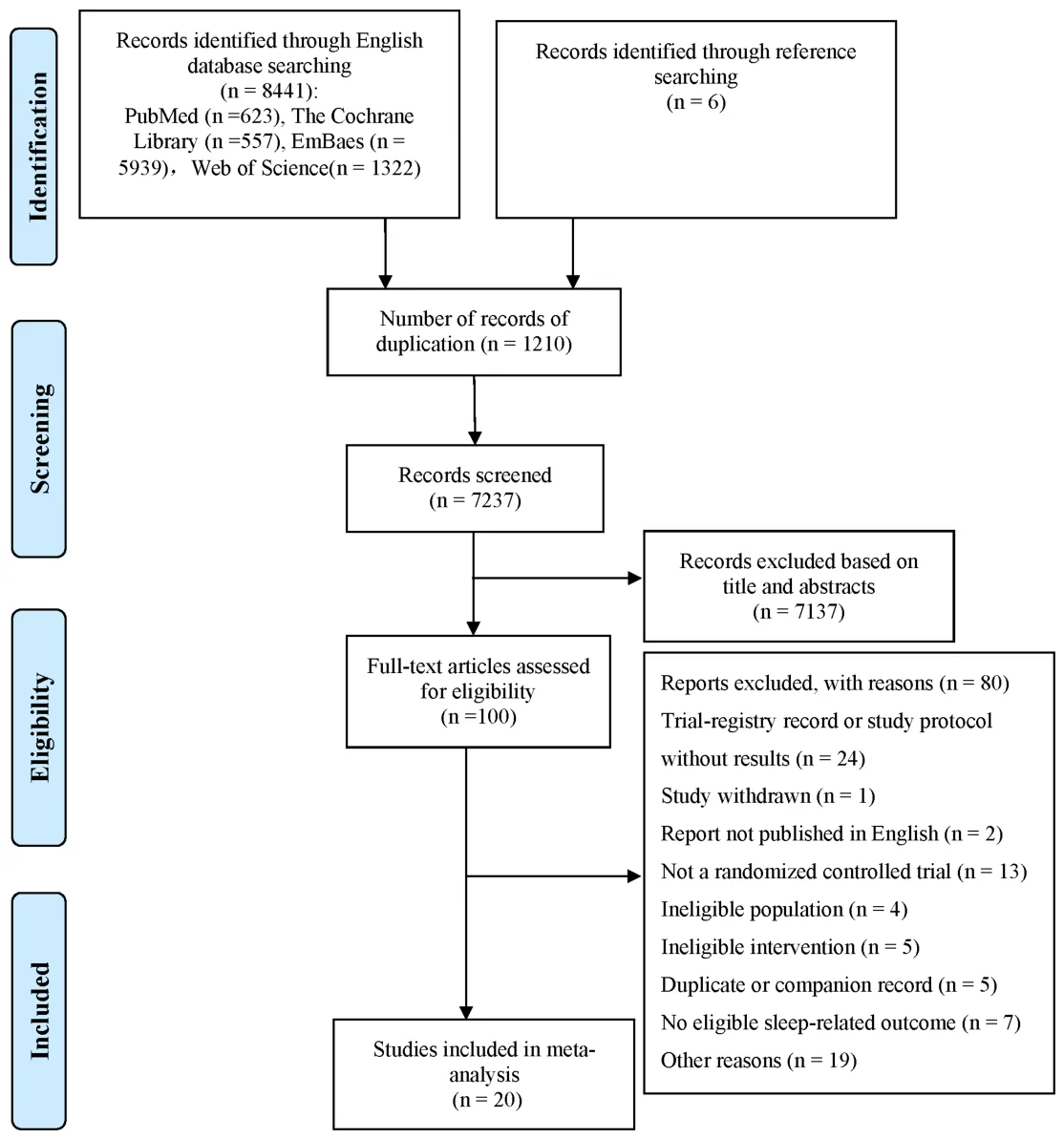
Preferred Reporting Items for Systematic Reviews and Meta-Analyses (PRISMA) 2020 flow diagram of the study-selection process.

This systematic review was reported in accordance with the Preferred Reporting Items for Systematic Reviews and Meta-Analyses (PRISMA) 2020 statement [7]. The study-selection process is presented in Figure 1, and the completed PRISMA 2020 checklist is provided as Supplementary File S1. Protocol registration: PROSPERO CRD420251123449.

### 2.2. Eligibility Criteria

Studies meeting the following criteria were included in this meta-analysis:

(1) Study type: RCTs.
(2) Participants: Adults aged ≥18 years with ESRD receiving hemodialysis or peritoneal dialysis.
(3) Sleep-related outcomes: Studies reported at least one eligible sleep-related outcome assessing sleep quality or insomnia symptom severity using a validated scale or a prespecified quantitative sleep measure, including the Pittsburgh Sleep Quality Index (PSQI), Insomnia Severity Index (ISI), Athens Insomnia Scale (AIS), sleep diaries, or a visual analog scale (VAS) for sleep disturbance.
(4) Interventions: At least one study arm received a non-pharmacological intervention lasting more than one week. Non-pharmacological interventions were defined as behavioral, psychological, exercise-based, physical, or complementary therapeutic approaches whose principal mechanism did not involve systemic administration of a pharmacological agent.
(5) Comparators: Comparator conditions included non-active controls (usual care or no intervention), sham/placebo controls, and active controls.
(6) Outcome measures: The primary outcome comprised post-intervention sleep quality or insomnia symptom severity, assessed using PSQI, ISI, AIS, or VAS. Secondary outcomes comprised post-intervention anxiety symptoms, depressive symptoms, and fatigue symptoms.

The prespecified study-design criterion was restricted to RCTs. One quasi-cluster randomized trial (Nakamura-Taira et al. [8]) was retained as a documented protocol deviation. This study used alternate allocation of four dialysis-schedule clusters and was therefore classified separately from the RCTs. Its effective sample size was corrected for clustering, and a sensitivity analysis excluding this study was performed. The population eligibility criterion applied during study selection was broader than that prespecified in the PROSPERO record because studies enrolling general dialysis populations were eligible if they reported an eligible sleep-related outcome, even when a sleep-related problem was not required at enrollment. This broadening is reported as a protocol deviation.

### 2.3. Exclusion Criteria

The following exclusion criteria were applied:

(1) Studies not published in English.
(2) Abstract-only reports, including conference abstracts, and study protocols.
(3) Participants who had undergone kidney transplantation.
(4) Retracted reports.

### 2.4. Data Sources and Search Strategy

A comprehensive systematic search was conducted in PubMed, Embase, the Cochrane Library, and Web of Science from inception to February 2026. The search strategy combined Medical Subject Headings (MeSH), Emtree terms, and free-text terms related to kidney disease, dialysis, renal replacement therapy, insomnia, sleep disturbance, sleep quality, and randomized trials. Key terms included “Kidney Diseases”, “Renal Dialysis”, “Peritoneal Dialysis”, “Renal Replacement Therapy”, “Sleep Initiation and Maintenance Disorders”, “insomnia”, “sleep quality”, “hemodialysis”, “peritoneal dialysis”, and “randomized controlled trial”. The complete search strategies for all databases are provided in Supplementary File S2. To ensure completeness, we manually screened the reference lists of all included articles and relevant systematic reviews.

### 2.5. Study Selection Process

The screening process involved:

(1) Independent title/abstract and full-text evaluation by two trained investigators (Huang and Ren) using predefined inclusion and exclusion criteria.
(2) Resolution of discrepancies through structured consensus meetings.
(3) Arbitration by a senior researcher (Zhou) for persistent disagreements.

### 2.6. Data Extraction

Data were extracted independently by two trained researchers (Huang and Ren) using a standardized procedure. Extracted information included:

(1) Study characteristics (author and publication year).
(2) Participant characteristics (population, sample size, age, sex, dialysis modality and dialysis frequency).
(3) Intervention and comparator details (type, duration, frequency, and delivery mode).
(4) Outcome measures.

For each outcome, baseline and post-intervention scores were extracted. The primary time point was immediately after completion of the intervention; when multiple post-intervention assessments were reported, the first assessment after intervention completion was used for the primary synthesis. Post-intervention scores were synthesized because they were the most consistently reported metric across studies. Data were recorded using predefined forms and verified by dual entry. Discrepancies were resolved through source-document review and discussion, with unresolved cases adjudicated by a senior researcher (Zhou).

### 2.7. Risk of Bias Assessment

The risk of bias for sleep-related outcomes and anxiety symptoms, depressive symptoms, and fatigue symptoms was independently assessed by two trained assessors (Huang and Ren) using RoB 2 [9]. For each included outcome, the assessment focused on the specific result—defined by the outcome, comparison, and time point—included in the corresponding meta-analysis. The following five domains were evaluated:

(1) Bias arising from the randomization process.
(2) Bias due to deviations from intended interventions.
(3) Bias due to missing outcome data.
(4) Bias in measurement of the outcome.
(5) Bias in selection of the reported result.

Each domain and the overall risk of bias were judged as “low risk,” “some concerns,” or “high risk.” For self-reported outcomes, participants were considered the outcome assessors when evaluating bias in outcome measurement. Discrepancies were resolved through discussion, with persistent disagreements adjudicated by a senior methodologist (Zhou).

### 2.8. Data Synthesis and Statistical Analysis

Continuous outcomes measured using different scales were pooled as SMDs calculated using Hedges’ *g*, with 95% CIs [10]; negative values favored the intervention group. The first assessment after completion of the intervention was used for the primary analysis. All outcomes were analyzed using REML–HKSJ random-effects models [11]. Between-study heterogeneity was assessed using Cochran’s Q, *I*^2^, and *τ*^2^, and 95% PIs were calculated when estimable [12].

For Nakamura-Taira et al. [8], the unit of allocation was the dialysis-schedule cluster rather than the individual participant. Four clusters defined by hospital and dialysis schedule were allocated by alternation. Clustering was addressed using the reported intracluster correlation coefficient of 0.02 and a design effect of 1.229. The effective sample size was reduced from 21 to 17 participants per group, while the group means and standard deviations remained unchanged.

Exploratory subgroup analyses were conducted according to intervention type, sleep-related eligibility criteria, and comparator type, with subgroup differences evaluated using random-effects Q-between tests. Robustness was assessed using leave-one-out sensitivity analysis. Funnel plots, Egger’s test, Begg’s test, and the trim-and-fill method were used to explore small-study effects and publication bias [13]. Statistical analyses were performed using R (version 4.6; https://www.r-project.org/) with the meta package (https://CRAN.R-project.org/package=meta (accessed on 6 September 2026)). All tests were two-sided, with *p* < 0.05 indicating statistical significance. Intervention-type subgroup analyses were restricted to intervention categories represented by at least two studies; categories represented by a single study remained in the primary analysis but were not included in the formal subgroup comparison.

Sensitivity analyses were conducted under three restricted study-selection scenarios. First, the synthesis was restricted to studies that required a diagnosis of insomnia, insomnia symptoms, a validated sleep-scale cutoff, or an explicit sleep complaint, sleep disturbance, or poor sleep quality at enrollment. Second, studies using active comparators were excluded, thereby retaining studies with non-active or sham/placebo comparators. Third, studies judged to be at high overall risk of bias were excluded, thereby retaining studies with an overall judgment of some concerns. Within each restricted dataset, a leave-one-out analysis was additionally performed to assess the influence of individual studies.

### 2.9. Certainty of Evidence Assessment

The certainty of evidence for sleep-related outcomes and anxiety symptoms, depressive symptoms, and fatigue symptoms was assessed using the Grading of Recommendations Assessment, Development and Evaluation (GRADE) approach [14]. The certainty of the evidence was initially rated as high because the body of evidence consisted predominantly of randomized trials and was subsequently evaluated for risk of bias, inconsistency, indirectness, imprecision, and publication bias. Certainty was rated as high, moderate, low, or very low. Detailed domain judgments and reasons for downgrading are presented in Supplementary File S3.

## 3. Results

### 3.1. Study Selection

Our systematic searches of PubMed, Embase, the Cochrane Library, and Web of Science through February 2026 identified 8441 records, and six additional records were identified through reference-list searching. After 1210 duplicates were removed using EndNote 20, 7237 unique records underwent title and abstract screening. Two reviewers independently screened these records against the predefined eligibility criteria, and 100 reports were assessed for eligibility. Eighty reports were excluded for documented reasons, leaving 20 studies for inclusion in the systematic review and meta-analysis. The study-selection process and reasons for exclusion are presented in the PRISMA 2020 flow diagram (Figure 1).

### 3.2. General Characteristics of the Studies Included in the Meta-Analysis

This systematic review included 19 RCTs and one quasi-cluster randomized trial [8,15,16,17,18,19,20,21,22,23,24,25,26,27,28,29,30,31,32,33]. The primary meta-analysis comprised an effective sample size of 1294 participants with ESRD; approximately 52% were female, and the mean age was 53.9 years. Sleep-related outcomes were assessed primarily using the PSQI, with other instruments including the ISI, AIS, and VAS. The key characteristics of the included studies are summarized in Table 1. Detailed information on participant characteristics, dialysis regimens, interventions, comparators, and outcome measures is provided in Supplementary File S4.

### 3.3. Risk of Bias

**Figure 2:**
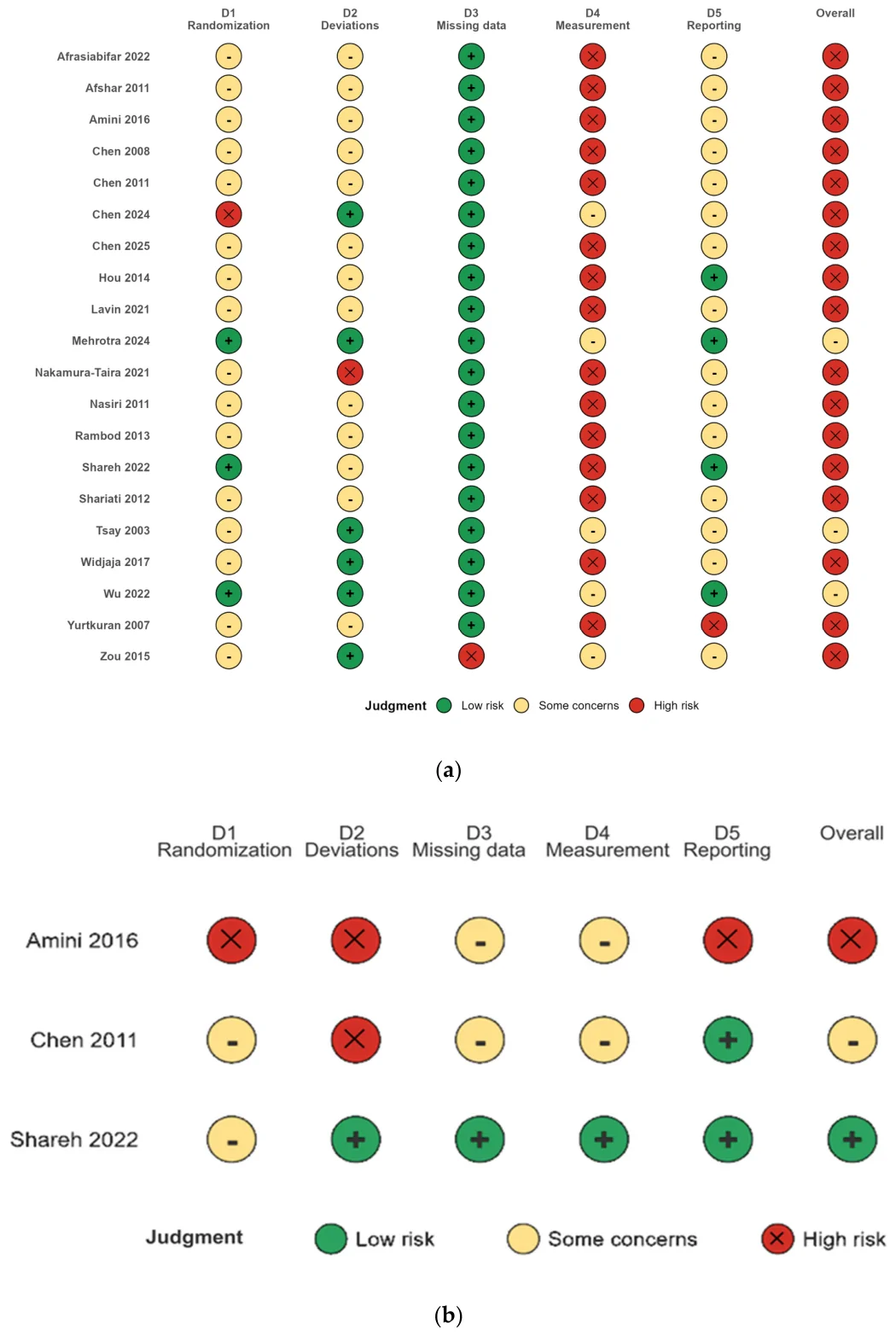

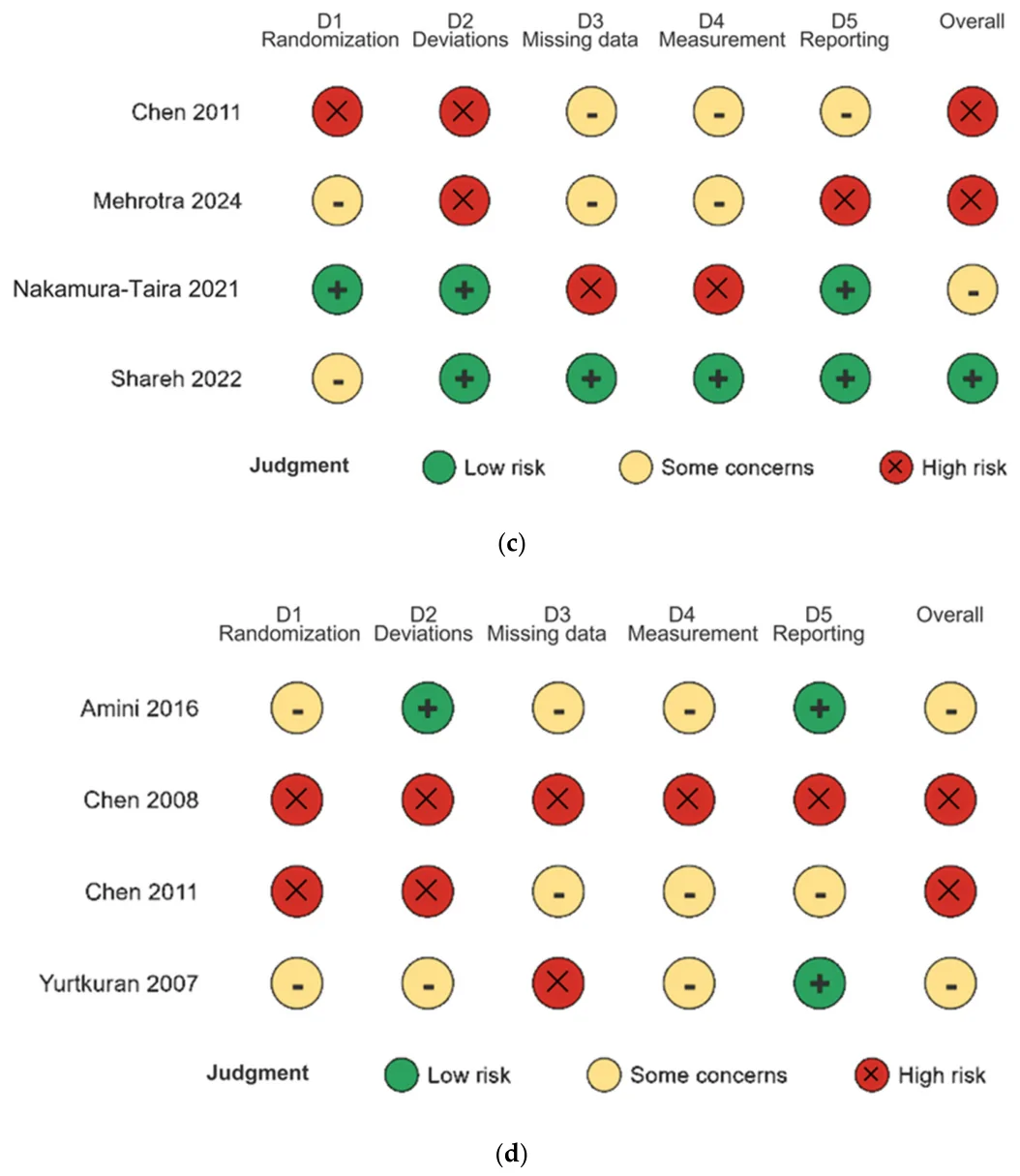
(**a**) Risk of bias assessment of sleep-related outcomes for included studies [8,15,16,17,18,19,20,21,22,23,24,25,26,27,28,29,30,31,32,33]. (**b**) Risk of bias assessment of anxiety symptoms for included studies [21,27,31]. (**c**) Risk of bias assessment of depressive symptoms for included studies [8,19,21,27]. (**d**) Risk of bias assessment of fatigue symptoms for included studies [15,25,27,31]. Green (+), yellow (−), and red (×) symbols indicate low risk, some concerns, and high risk of bias, respectively.

The RoB 2 assessment of sleep-related outcomes indicated an overall high risk of bias in 17 studies (85.0%) and some concerns in three studies (15.0%); no study was judged to be at low overall risk of bias (Figure 2a). Bias in outcome measurement was the principal concern, with 15 studies (75.0%) judged to be at high risk, largely because subjective sleep outcomes were self-reported by participants who could identify their assigned interventions. In contrast, 19 studies (95.0%) were judged to be at low risk for missing outcome data. Most studies raised some concerns regarding the randomization process, deviations from intended interventions, and selection of the reported result.

For anxiety symptoms, the overall judgments were low risk for one study, some concerns for one study, and high risk for one study. For depressive symptoms, the overall judgments were low risk for one study, some concerns for one study, and high risk for two studies. For fatigue symptoms, the overall judgments were some concerns for two studies and high risk for two studies; no study was judged to be at low overall risk of bias. Bias due to deviations from intended interventions was the domain most frequently judged to be at high risk across these secondary outcomes (Figure 2b–d). Detailed judgments and supporting rationales are provided in Supplementary File S6. The certainty of evidence for sleep-related outcomes, anxiety symptoms, depressive symptoms, and fatigue symptoms was rated as very low because of very serious risk of bias and, depending on the outcome, inconsistency and/or imprecision (Supplementary File S3).

## 4. Outcome Analysis

### 4.1. Main Analysis

#### 4.1.1. Sleep-Related Outcomes

**Figure 3:**
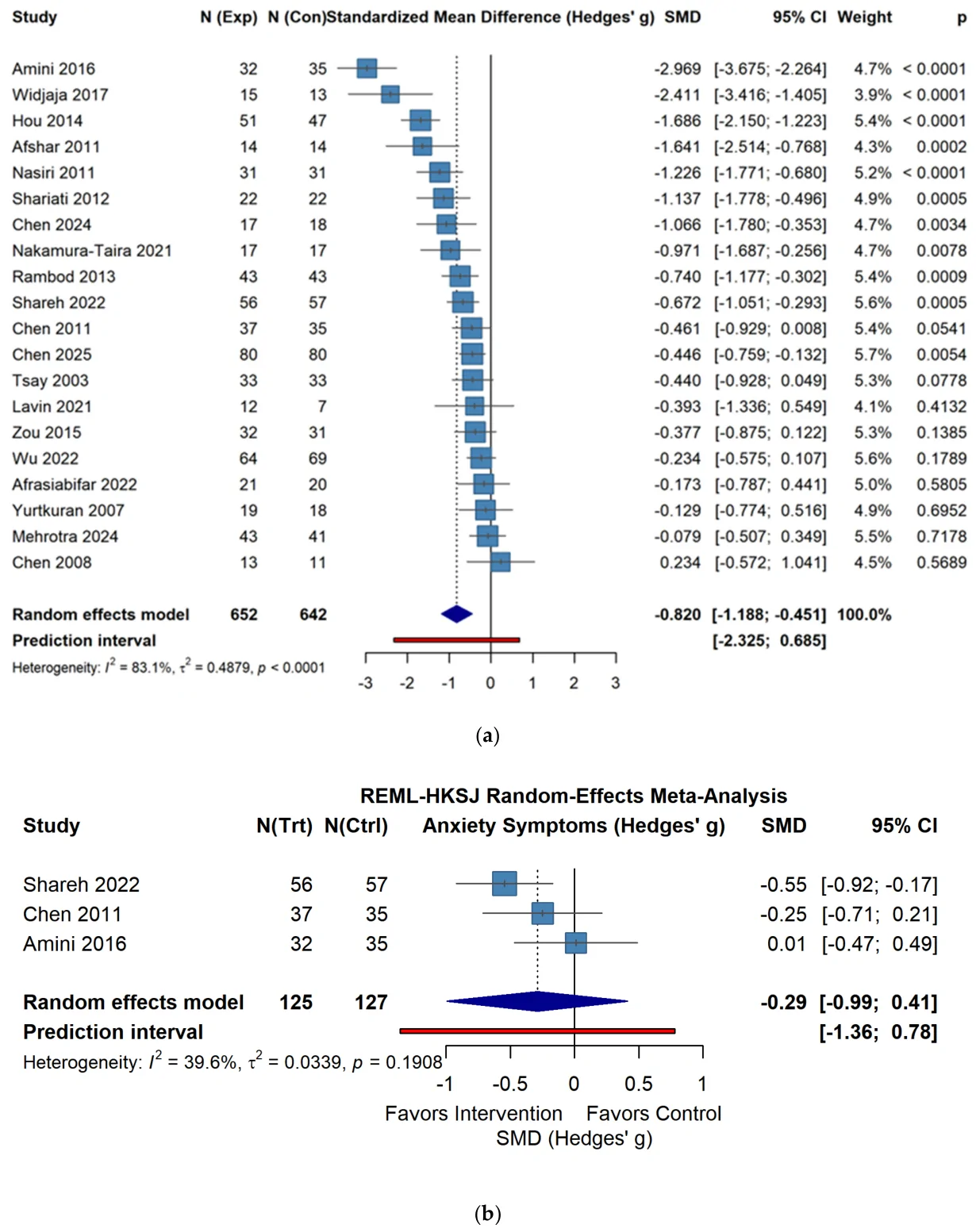

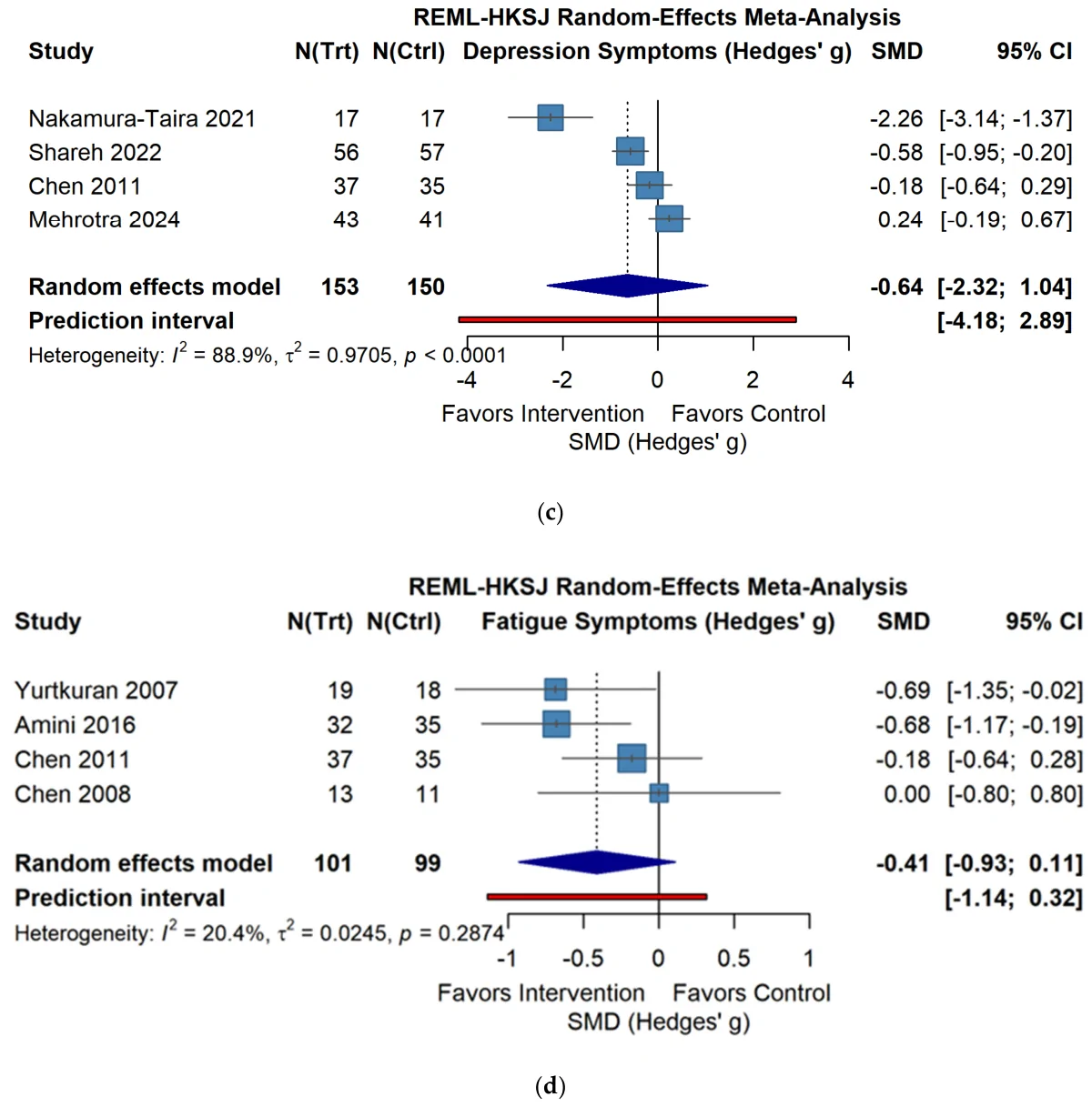
(**a**) Forest plot of the effects of non-pharmacological interventions on sleep-related outcomes [8,15,16,17,18,19,20,21,22,23,24,25,26,27,28,29,30,31,32,33]. (**b**) Anxiety symptoms [21,27,31]. (**c**) Depressive symptoms [8,19,21,27]. (**d**) Fatigue symptoms [15,25,27,31]. Blue squares with horizontal lines show individual estimates and 95% confidence intervals (CIs); diamonds show pooled estimates and 95% CIs. Red bars show 95% prediction intervals. Solid vertical lines mark no effect; dotted lines mark pooled estimates. SMD, standardized mean difference; N, sample size; Exp/Trt, intervention; Con/Ctrl, comparator; REML, restricted maximum likelihood; HKSJ, Hartung–Knapp–Sidik–Jonkman.

A pooled analysis of 20 trials, including one quasi-cluster randomized trial, yielded an effective sample size of 1294 after correction for clustering. The REML–HKSJ random-effects model favored non-pharmacological interventions over comparator conditions (Hedges’ *g* = −0.820, 95% CI −1.188 to −0.451; *p* = 0.0002), suggesting that these interventions may improve sleep quality or reduce insomnia symptom severity among adults with ESRD receiving dialysis. However, between-study heterogeneity was substantial (*I*^2^ = 83.1%; *τ*^2^ = 0.4879). The 95% PI ranged from −2.325 to 0.685 (Figure 3a).

#### 4.1.2. Anxiety Symptoms

Across three RCTs involving 252 participants, the REML–HKSJ random-effects model showed no statistically significant effect of non-pharmacological interventions on anxiety symptoms (Hedges’ *g* = −0.29, 95% CI −0.99 to 0.41; *p* = 0.219). Between-study heterogeneity was moderate (*I*^2^ = 39.6%; *τ*^2^ = 0.0339; Cochran’s Q test, *p* = 0.1908). The 95% PI ranged from −1.36 to 0.78 (Figure 3b).

#### 4.1.3. Depressive Symptoms

Four studies were included in the analysis of depressive symptoms, with a total effective sample size of 303 after correction for the clustered design of Nakamura-Taira et al. [8]. The pooled effect of non-pharmacological interventions on depressive symptoms was not statistically significant (Hedges’ *g* = −0.64, 95% CI −2.32 to 1.04; *p* = 0.313). Between-study heterogeneity was substantial (*I*^2^ = 88.9%; *τ*^2^ = 0.9705; Cochran’s Q test, *p* < 0.0001). The 95% PI ranged from −4.18 to 2.89 (Figure 3c).

#### 4.1.4. Fatigue Symptoms

Four RCTs involving 200 participants were included in the analysis of fatigue symptoms. The pooled estimate from the REML–HKSJ random-effects model suggested that non-pharmacological interventions might improve fatigue symptoms; however, the difference did not reach statistical significance (Hedges’ *g* = −0.41, 95% CI −0.93 to 0.11; *p* = 0.087). Between-study heterogeneity was low (*I*^2^ = 20.4%; *τ*^2^ = 0.0245; Cochran’s Q test, *p* = 0.2874). The 95% PI ranged from −1.14 to 0.32 (Figure 3d).

### 4.2. Subgroup Analyses

To investigate sources of heterogeneity, we conducted exploratory subgroup analyses stratified by intervention type, sleep-related eligibility criteria, and comparator type.

#### 4.2.1. Intervention Type

**Figure 4:**
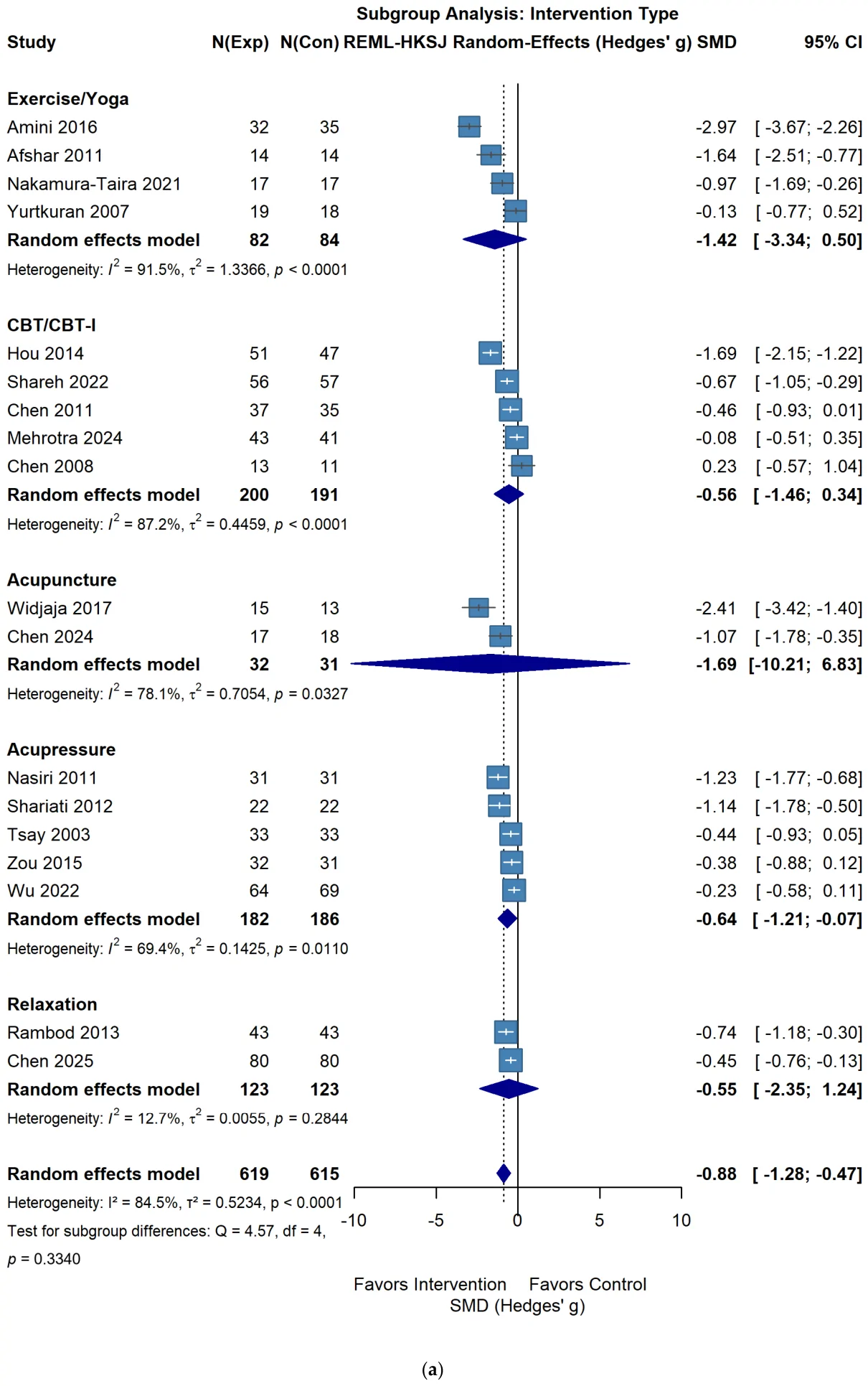

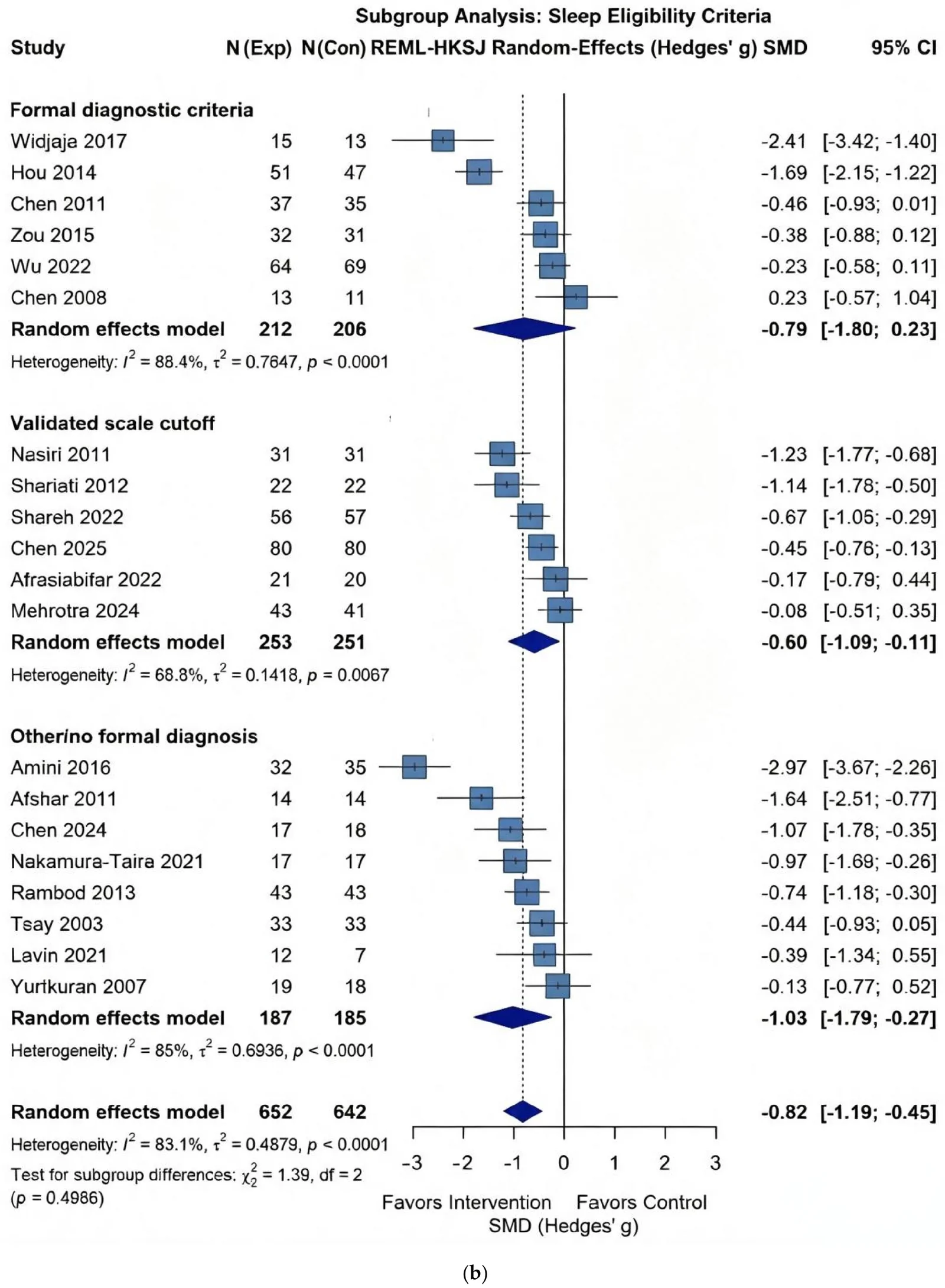

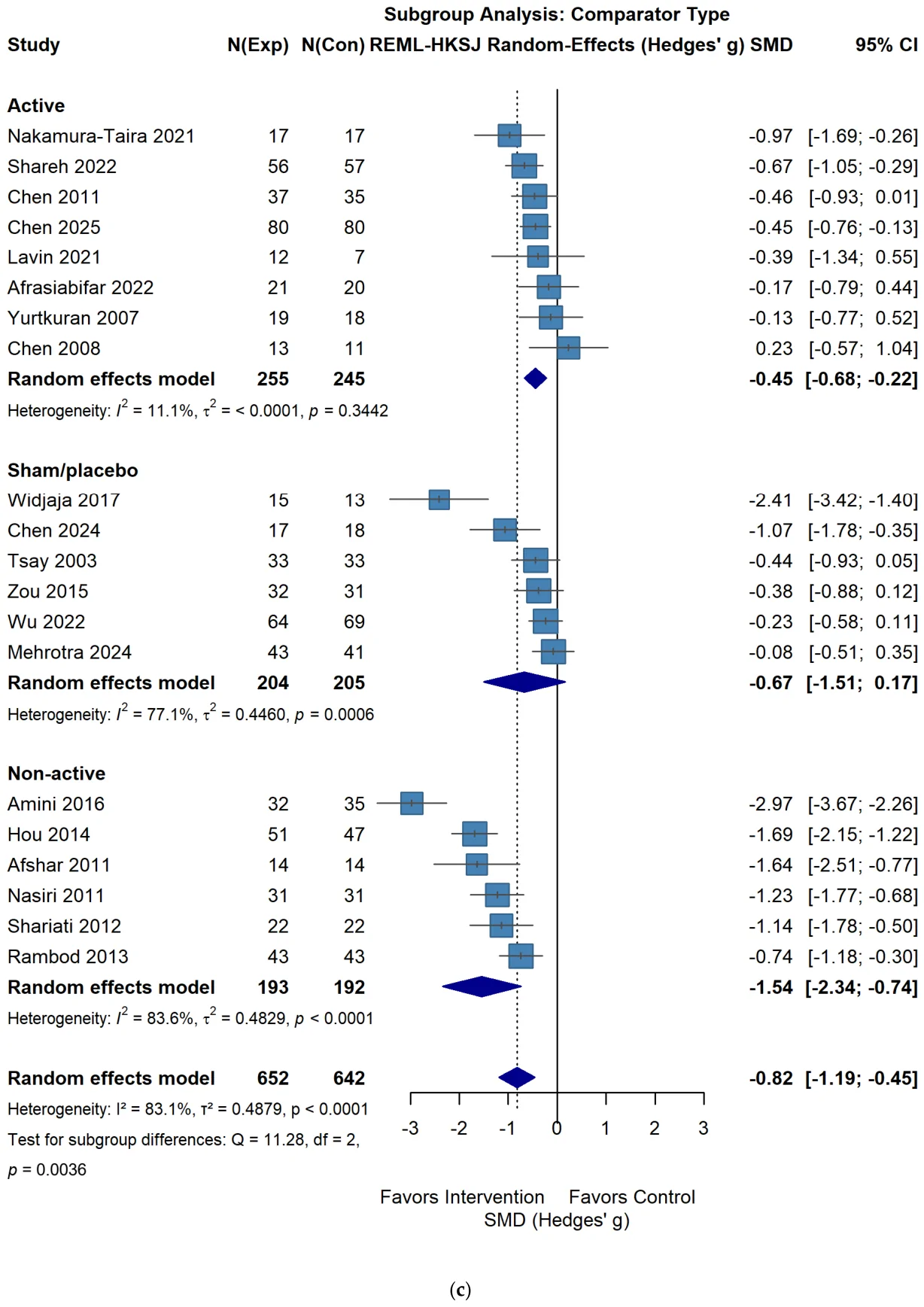
(**a**) Forest plot of the intervention-type subgroup analysis for sleep-related outcomes [8,15,16,17,19,20,21,22,23,24,25,26,27,28,29,31,32,33]. (**b**) Forest plot of the sleep-related eligibility subgroup analysis [8,15,16,17,18,19,20,21,22,23,24,25,26,27,28,29,30,31,32,33]. (**c**) Forest plot of the comparator-type subgroup analysis [8,15,16,17,18,19,20,21,22,23,24,25,26,27,28,29,30,31,32,33]. Blue squares with horizontal lines show individual estimates and 95% CIs; diamonds show pooled estimates and 95% CIs. Solid vertical lines mark no effect; dotted lines mark overall pooled estimates.

Eighteen of the 20 studies were included in the intervention-type subgroup analysis. Mindfulness and footbath were retained in the primary analysis but were not included in the formal subgroup comparison because each category contained only one study. The formal test showed no statistically significant difference among the five analyzed intervention categories (Q-between = 4.57, df = 4, *p* = 0.3340). These findings do not support intervention type as a major source of the observed between-study heterogeneity, although the subgroup analysis should be interpreted as exploratory (Figure 4a).

#### 4.2.2. Sleep-Related Eligibility Criteria

All 20 trials were included in the sleep-related eligibility subgroup analysis. Six studies used formal diagnostic criteria, six used a validated scale cutoff, and eight were classified as other/no formal diagnosis. The formal test showed no statistically significant difference among the three subgroups (Q-between = 1.39, df = 2, *p* = 0.4986). These findings do not support the method used to define sleep-related eligibility as a major source of the observed between-study heterogeneity (Figure 4b).

#### 4.2.3. Comparator Type

All 20 trials were included in the comparator-type subgroup analysis. Eight studies used active comparators, six used sham/placebo comparators, and six used non-active comparators. The random-effects Q-between test showed a statistically significant difference in effect estimates among the three comparator-type subgroups (Q-between = 11.28, df = 2, *p* = 0.0036), supporting comparator type as a potentially important source of the observed between-study heterogeneity (Figure 4c).

### 4.3. Publication Bias

**Figure 5:**
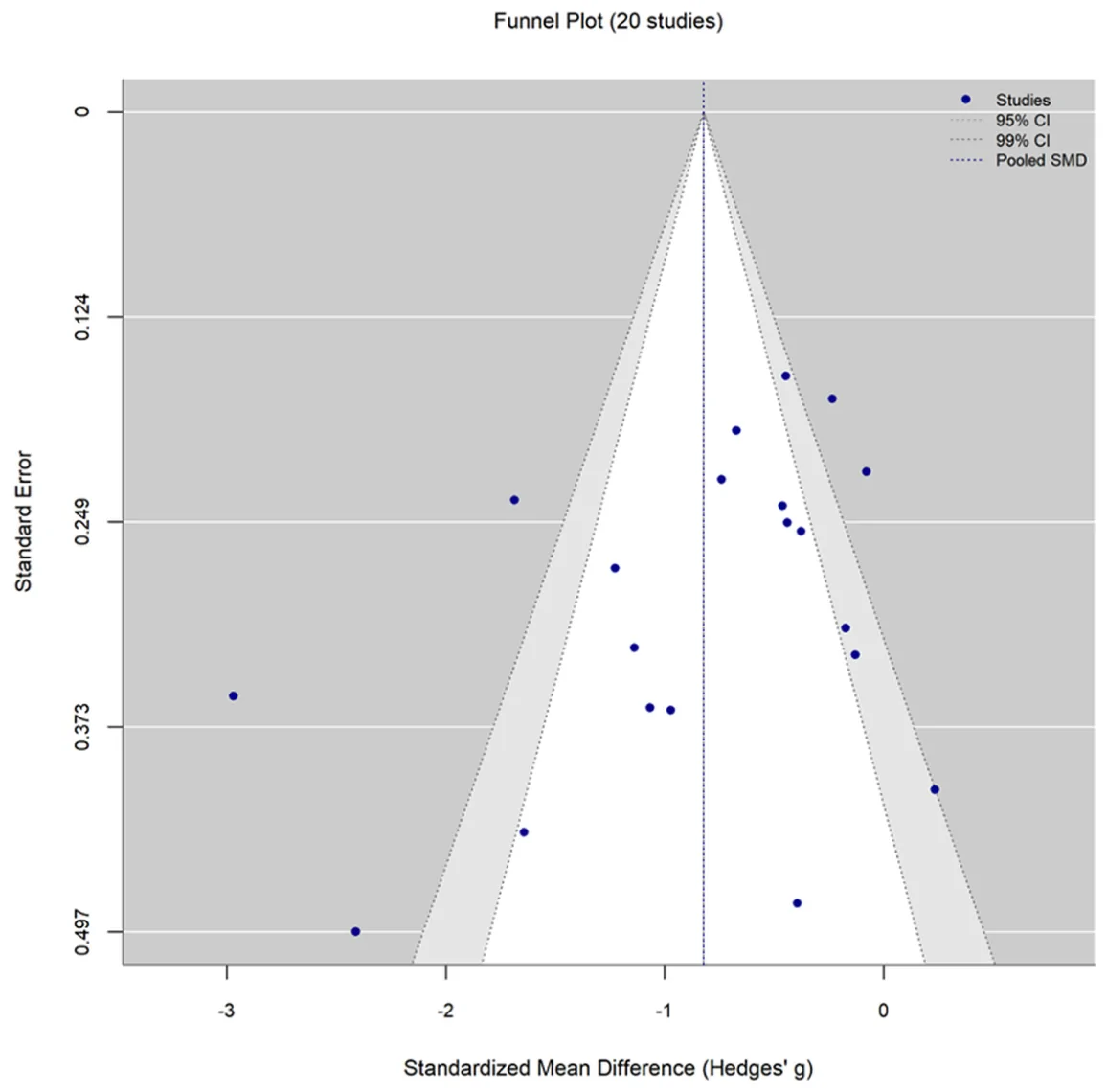
Funnel plot for the primary sleep-related outcome. Blue points represent individual studies, and the dotted vertical line indicates the pooled SMD. The white region is within the 95% pseudo-confidence limits; the light-gray region lies between the 95% and 99% limits, and the darker-gray region lies outside the 99% limits.

To evaluate potential small-study effects and publication bias, we used a funnel plot, Egger’s regression test, Begg’s rank-correlation test, and the trim-and-fill method. The funnel plot appeared asymmetric (Figure 5). However, neither Egger’s test (*t* = −1.841, df = 18, *p* = 0.082) nor Begg’s test (Kendall’s *τ* = −0.232, *p* = 0.165) was statistically significant (Table 2). The trim-and-fill method imputed no additional studies, and the pooled estimate generated within that analysis remained unchanged (Hedges’ *g* = −0.823). This estimate was calculated separately from the primary REML–HKSJ estimate (Hedges’ *g* = −0.820). These analyses provided no statistically significant evidence of small-study effects. However, given the substantial heterogeneity (*I*^2^ = 83.1%), funnel-plot asymmetry may also reflect differences in interventions, participant characteristics, comparator designs, or study quality.

### 4.4. Sensitivity Analysis

#### 4.4.1. Leave-One-Out Sensitivity Analysis

**Figure 6:**
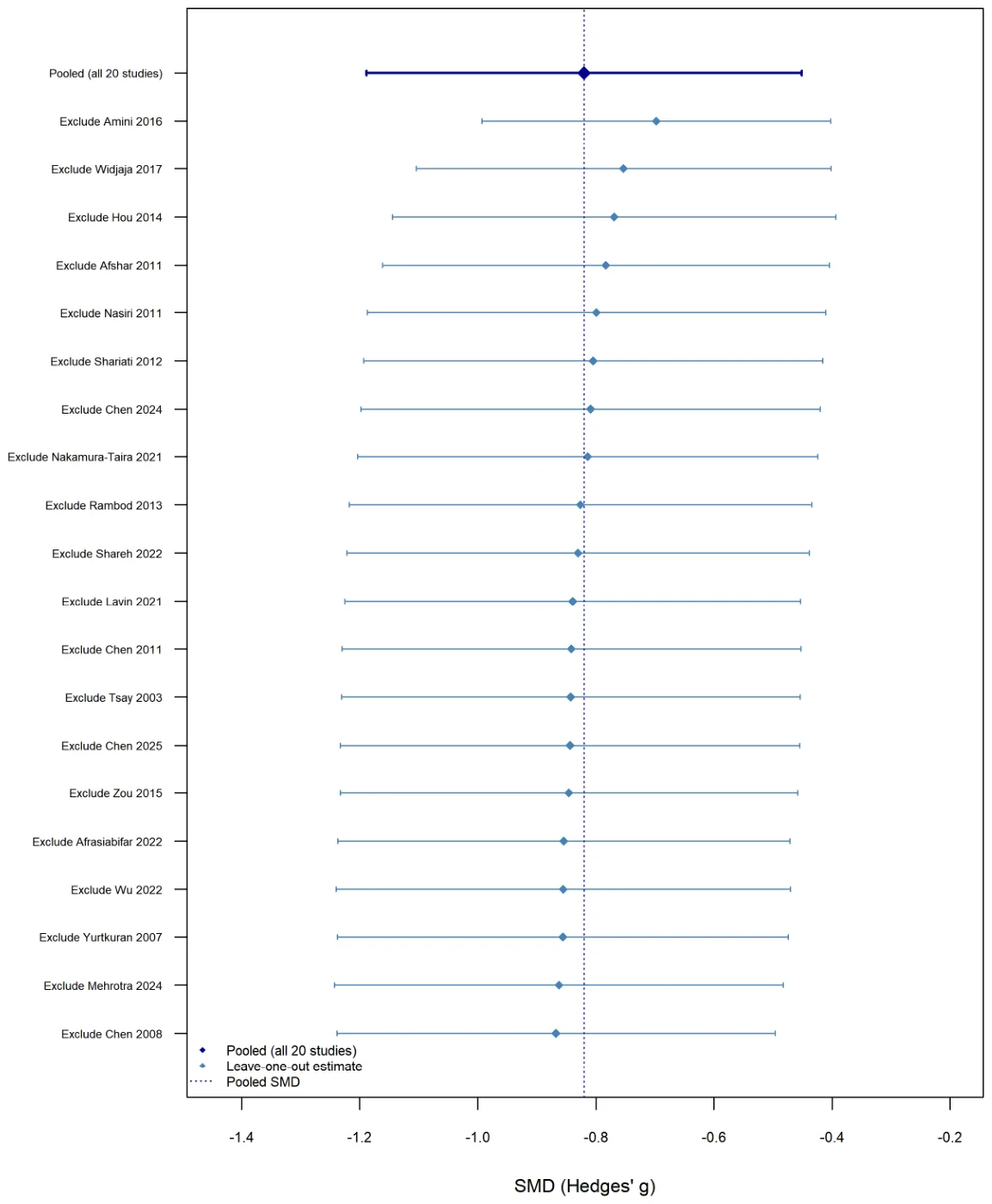
Leave-one-out sensitivity analysis of the primary sleep-related outcome [8,15,16,17,18,19,20,21,22,23,24,25,26,27,28,29,30,31,32,33]. Dark blue shows the pooled estimate and 95% CI for all 20 studies; light blue shows estimates and 95% CIs after omitting each study in turn. The dotted vertical line marks the pooled estimate for all studies.

Sensitivity analysis based on the leave-one-out method showed stable results. The pooled Hedges’ *g* estimates ranged from −0.868 to −0.698, remained consistently negative, and were statistically significant after the exclusion of each individual study (all *p* < 0.001). Thus, no individual study materially affected the overall estimate (Hedges’ *g* = −0.820) (Figure 6).

#### 4.4.2. Methodological Sensitivity Analyses

Restricting the synthesis to the 14 studies that required a sleep-related eligibility criterion at enrollment yielded a pooled effect favoring non-pharmacological interventions (Hedges’ *g* = −0.713, 95% CI −1.109 to −0.317; *p* = 0.0019). Substantial heterogeneity remained (*I*^2^ = 80.2%; *τ*^2^ = 0.2488; Cochran’s Q = 65.53, df = 13; *p* < 0.0001). In the leave-one-out analysis, pooled estimates ranged from −0.769 to −0.615, and all 95% CIs excluded zero, indicating that the restricted pooled result was not materially influenced by any individual study (Supplementary File S7a).

After studies using active comparators were excluded, 12 studies with non-active or sham/placebo comparators remained. The pooled effect favored non-pharmacological interventions (Hedges’ *g* = −1.116, 95% CI −1.672 to −0.559; *p* = 0.0010), although substantial heterogeneity remained (*I*^2^ = 88.3%; *τ*^2^ = 0.5489; Cochran’s Q = 94.03, df = 11; *p* < 0.0001). In the leave-one-out analysis, pooled estimates ranged from −1.217 to −0.929, and all 95% CIs excluded zero. Thus, the pooled result after excluding active comparators was not materially influenced by any individual study (Supplementary File S7b).

After the 17 studies judged to be at high overall risk of bias were excluded, three studies with an overall judgment of some concerns remained. The pooled effect was not statistically significant (Hedges’ *g* = −0.235, 95% CI −0.631 to 0.161; *p* = 0.1252), with no evidence of between-study heterogeneity (*I*^2^ = 0.0%; *τ*^2^ = 0; Cochran’s Q = 1.19, df = 2; *p* = 0.5529). In the leave-one-out analysis, pooled estimates ranged from −0.302 to −0.174, and all 95% CIs crossed zero. Therefore, statistical significance was not maintained after studies at high overall risk of bias were excluded; however, this finding should be interpreted cautiously because only three studies remained (Supplementary File S7c).

After Nakamura-Taira et al. [8] was excluded, the sensitivity analysis of the remaining 19 studies yielded a pooled effect favoring non-pharmacological interventions (Hedges’ *g* = −0.807, 95% CI −1.194 to −0.421; *p* = 0.0004). Substantial heterogeneity remained (*I*^2^ = 83.9%; *τ*^2^ = 0.3588; Cochran’s Q = 111.92, df = 18; *p* < 0.0001). In the leave-one-out analysis within this restricted dataset, pooled estimates ranged from −0.857 to −0.684, and all 95% CIs excluded zero. The direction and magnitude of the pooled effect were consistent with the primary analysis of all 20 studies (Hedges’ *g* = −0.820), indicating that exclusion of Nakamura-Taira et al. [8] did not materially alter the overall result (Supplementary File S7d).

## 5. Discussion

This updated systematic review and meta-analysis synthesized evidence from studies evaluating the effects of non-pharmacological interventions on sleep-related outcomes, anxiety symptoms, depressive symptoms, and fatigue symptoms in patients with dialysis-dependent ESRD. The pooled results suggested that non-pharmacological interventions may improve sleep-related outcomes; however, no statistically significant improvements were observed in anxiety symptoms, depressive symptoms, or fatigue symptoms.

Compared with the previous review [6], this updated review included ten trials published after 2015, reflecting sustained research interest in non-pharmacological approaches to improving sleep-related outcomes in patients with dialysis-dependent ESRD. Our finding that these interventions may improve sleep-related outcomes is clinically relevant because polypharmacy and a high medication burden are common in this population and may contribute to impaired quality of life and medication-related adverse events [34]. Non-pharmacological interventions may therefore provide a useful approach to managing sleep problems without further increasing medication burden.

The evidence underlying these potential benefits encompassed seven categories of non-pharmacological interventions: exercise, acupressure, cognitive behavioral therapy (CBT), acupuncture, footbath, mindfulness-based interventions, and relaxation-based interventions. Across these diverse approaches, the pooled analysis suggested a potential improvement in sleep-related outcomes. However, the interventions differed substantially in their mechanisms, intensity, duration, and comparator conditions; therefore, the overall effect estimate should not be interpreted as evidence that each intervention category is effective. No statistically significant improvements were observed in anxiety symptoms, depressive symptoms, or fatigue symptoms. These findings may be related to the small number of studies reporting these outcomes, limited sample sizes, short intervention or follow-up periods, and the fact that most interventions primarily targeted sleep rather than these associated symptoms.

We further explored whether the effect estimates varied according to intervention type. Mindfulness and footbath interventions yielded Hedges’ *g* values of −0.39 and −0.17, respectively [18,30]; however, because each intervention was represented by only one study, neither was included in the formal test for subgroup differences. Among the intervention types included in the formal comparison, the pooled estimate for acupressure favored the intervention group (Hedges’ *g* = −0.64, 95% CI −1.21 to −0.07). The direction of this finding was broadly consistent with the meta-analysis by Yang et al., which suggested that acupressure may improve sleep quality in patients receiving dialysis [6]. Another meta-analysis evaluating auricular acupressure for sleep disturbances in patients undergoing maintenance hemodialysis also reported a beneficial effect (mean difference = −1.97, 95% CI −2.62 to −1.32; *p* < 0.0001) [35]. However, substantial heterogeneity remained within the acupressure subgroup (*I*^2^ = 69.4%; *τ*^2^ = 0.1425; Cochran’s Q test, *p* = 0.0110), and the formal test for differences among intervention types was not statistically significant (Q-between = 4.57, df = 4; *p* = 0.3340). Therefore, although the within-subgroup estimate favored acupressure, the available evidence does not demonstrate that acupressure is more effective than the other intervention types.

For sleep-related eligibility criteria, the overall subgroup analysis showed no statistically significant difference in effect estimates among the three subgroups. Studies relying solely on validated scale cutoffs may have included a broader and more clinically heterogeneous population, whereas studies using formal diagnostic criteria may have enrolled patients with more clearly characterized insomnia. Although differences in baseline symptom severity and clinical characteristics may have influenced the effect estimates, the available data were insufficient to determine the direction or magnitude of these influences. The point estimates in these two subgroups favored non-pharmacological interventions, but substantial residual heterogeneity remained within the subgroups. Thus, the available evidence does not demonstrate that the criteria used to identify eligible sleep problems modify the intervention effect or adequately explain the observed heterogeneity.

For comparator type, heterogeneity varied across comparator subgroups. Compared with the overall heterogeneity (*I*^2^ = 83.1%), heterogeneity was low in the active-comparator subgroup (*I*^2^ = 11.1%) but remained substantial in the sham/placebo-comparator subgroup (*I*^2^ = 77.1%) and the non-active-comparator subgroup (*I*^2^ = 83.6%). This pattern suggests that comparator design may contribute to between-study heterogeneity, although this interpretation should be considered alongside the formal test for subgroup differences.

Anxiety and depression are common among patients receiving dialysis, with pooled prevalence estimates of approximately 31% and 40%, respectively [36]. Both pharmacological and non-pharmacological approaches are used to manage these symptoms, and non-pharmacological interventions are of particular interest because they may avoid further increasing medication burden. However, our meta-analysis found no statistically significant improvements in anxiety or depression. This finding differs from that of Ng et al., who evaluated eight studies of CBT involving 540 hemodialysis patients with comorbid depression. Their analysis showed a significant reduction in depression (SMD = −0.68, 95% CI −0.94 to −0.42) and borderline evidence of improvement in anxiety (SMD = −0.99, 95% CI −1.99 to 0.00; *p* = 0.05) [37]. Similarly, a 2023 meta-analysis reported reductions in depression (SMD = −0.85, 95% CI −1.17 to −0.52) and anxiety (SMD = −0.99, 95% CI −1.65 to −0.33) [38]. These differences may reflect variations in the target populations and intervention objectives: the previous reviews focused on patients with depression or on interventions specifically designed to improve psychological outcomes, whereas most interventions in our review primarily targeted sleep. Moreover, the small number of studies reporting anxiety and depression, limited sample sizes, and short follow-up periods may have reduced the ability of our analysis to detect significant effects. Therefore, our findings should not be interpreted as evidence that non-pharmacological interventions have no effect on anxiety or depression, and further adequately powered trials specifically targeting these outcomes are needed.

Fatigue is one of the most common and debilitating symptoms experienced by patients receiving dialysis. Postdialysis fatigue, a specific form of fatigue, has been reported in 60–97% of patients receiving in-center hemodialysis [39]. A US mixed-methods study incorporating a nationally distributed survey also identified fatigue as a high-priority symptom for the development and evaluation of new interventions [40]. However, effective targeted strategies for managing fatigue remain limited. The certainty of previous evidence regarding non-pharmacological interventions also remains limited. Yang et al. pooled five RCTs and reported a significant improvement in fatigue (SMD = 0.66, 95% CI 0.44 to 0.88) [6]. A subsequent Cochrane review found low-certainty evidence that exercise, aromatherapy, massage, and acupressure may improve fatigue, whereas the effects of other interventions remained uncertain [41]. In our meta-analysis, the pooled effect estimate favored non-pharmacological interventions, but the CI included the null value, indicating that a statistically significant improvement in fatigue was not established. Differences between these findings may be related to variations in eligibility criteria, intervention types and durations, fatigue measurement instruments and assessment time points, and participants’ clinical characteristics.

This study has several limitations. First, among the 20 included studies, some were judged as having “some concerns” or a “high risk of bias” in one or more RoB 2 domains, which may reduce confidence in the pooled effect estimates. Second, this review included only reports published in English, potentially introducing language bias and resulting in the omission of relevant evidence. Third, because change scores and their corresponding variances were incompletely reported, post-intervention scores were used in the primary analysis. Although baseline comparability for the primary sleep outcome was examined, chance baseline imbalances in small trials may still have affected the between-group effect estimates. Finally, in the presence of substantial heterogeneity, funnel plots and related statistical tests have limited ability to detect small-study effects or publication bias. Therefore, the non-significant results of Egger’s and Begg’s tests and the absence of additional studies imputed by the trim-and-fill method cannot rule out publication bias.

## 6. Conclusions

This meta-analysis of randomized trials and one quasi-cluster randomized trial suggests that non-pharmacological interventions may improve sleep-related outcomes in patients receiving dialysis for ESRD. However, the certainty of evidence is very low, between-study heterogeneity is substantial, the prediction interval crosses the null, and most included studies are at high overall risk of bias. Current evidence does not demonstrate statistically significant improvements in anxiety symptoms, depressive symptoms, or fatigue symptoms and remains insufficient to support firm clinical recommendations. Further well-designed, adequately powered, and clinically homogeneous randomized trials are required to confirm the magnitude and consistency of any benefit.

## Figures and Tables

**Table 1:** Key characteristics of the included studies.

First Author	Year	Sample Size (Male), n	Age (Years)	Intervention (Arm)	Sleep-Related Outcome Measure(s)
Afrasiabifar et al. [30]	2022	62 (26)	All: 51.01 ± 13.91	Exp: Footbath Con: Effleurage	PSQI
Amini et al. [31]	2016	100 (36)	Exp: 54.31 Con: 55.22	Exp: Aerobic exercise Con: Usual care	PSQI
Chen et al. [15]	2008	24 (15)	All: 49.2 ± 12.0	Exp: CBT + sleep hygiene education Con: Sleep hygiene education only	PSQI
Chen et al. [16]	2024	35 (NA)	NA	Exp: Acupuncture Con: Sham acupuncture	ISI
Chen et al. [29]	2025	160 (73)	Exp: 36 Con: 44	Exp: CBT-I with JPMR Con: CBT-I	PSQI/ISI
Hou et al. [17]	2014	103 (42)	Exp: 54.5 ± 13.8 Con: 52.4 ± 14.5	Exp: CBT Con: Conventional HD	PSQI
Lavin et al. [18]	2021	19 (10)	All: 68.7 ± 6.4	Exp: Brief Mindfulness Intervention Con: Health Enhancement Program	AIS
Mehrotra et al. [19]	2024	126 (63)	Exp: 60.9 Con: 57.2	Exp: CBT-I Con: Placebo	ISI/PSQI
Nakamura-Taira et al. [8]	2021	42 (22)	Exp: 74.90 ± 2.23 Con: 72.57 ± 2.26	Exp: Low-intensity resistance exercise Con: Stretching	AIS
Rambod et al. [20]	2013	86 (53)	All: 49.89 ± 12.47	Exp: Benson’s relaxation technique Con: Routine care	PSQI
Shareh et al. [21]	2022	116 (69)	Exp: 43.7 ± 6.4 Con: 46.4 ± 7.9	Exp: CBGT-I Con: Psychoeducation	PSQI
Wu et al. [22]	2022	133 (63)	Exp: 52.3 ± 13.1 Con: 55.9 ± 13.3	Exp: Auricular acupressure Con: Sham auricular acupressure	PSQI
Widjaja et al. [32]	2017	28 (15)	Exp: 43.87 ± 10.98 Con: 49.54 ± 9.21	Exp: Acupuncture Con: Sham acupuncture	PSQI
Zou et al. [23]	2015	63 (28)	Exp: 53.28 ± 12.68 Con: 58.55 ± 10.00	Exp: Auricular acupressure Con: Sham acupressure	PSQI
Tsay et al. [24]	2003	98 (44)	All: 55.52 ± 12.98	Exp: Acupressure Con: Sham acupressure	PSQI
Yurtkuran et al. [25]	2007	37 (NA)	NA	Exp: Modified yoga Con: Range-of-motion exercises	VAS
Afshar et al. [26]	2011	28 (28)	Exp: 50.71 ± 21.06 Con: 53.00 ± 19.40	Exp: Intradialytic aerobic exercise Con: Usual care	PSQI
Chen et al. [27]	2011	72 (30)	All: 58 ± 11	Exp: CBT Con: Sleep hygiene education	PSQI
Nasiri et al. [33]	2011	62 (NA)	Exp: 48.68 ± 13.49 Con: 47.81 ± 11.55	Exp: Acupressure Con: Routine care	PSQI
Shariati et al. [28]	2012	44 (23)	Exp: 53.5 ± 12.5 Con: 55.5 ± 10.6	Exp: Acupressure Con: Routine care	PSQI

Abbreviations: AIS, Athens Insomnia Scale; CBGT-I, cognitive behavioral group therapy for insomnia; CBT, cognitive behavioral therapy; CBT-I, cognitive behavioral therapy for insomnia; Con, control group; Exp, experimental group; HD, hemodialysis; ISI, Insomnia Severity Index; JPMR, Jacobson progressive muscle relaxation; NA, not available; PSQI, Pittsburgh Sleep Quality Index; VAS, visual analog scale. Note: For multi-arm trials, Table 1 reports the total trial sample size, whereas the meta-analysis used only the prespecified pairwise comparison; unused arms were excluded to avoid double-counting.

**Table 2:** Statistical test results for publication bias and small-study effects.

Test Method	Statistic	*p* Value	Interpretation
Egger’s regression test	*t* = −1.841, df = 18	0.082	Not statistically significant; no clear evidence of small-study effects
Begg’s rank correlation test	Kendall’s *τ* = −0.232	0.165	Not statistically significant
Duval–Tweedie trim-and-fill analysis	Studies imputed = 0	—	No additional studies were imputed; the pooled estimate within the trim-and-fill analysis remained unchanged (Hedges’ *g* = −0.823)

## Data Availability

All data analyzed in this systematic review and meta-analysis were extracted from previously published studies. The extracted dataset is available from the corresponding author upon reasonable request.

## Supplementary Materials

The following supporting information can be downloaded at https://www.mdpi.com/article/10.3390/healthcare14182921/s1. Supplementary File S1: PRISMA 2020 checklist [7]. Supplementary File S2: Complete search strategies for all databases. Supplementary File S3: Detailed GRADE judgments [14]. Supplementary File S4: Detailed information on participant characteristics, dialysis regimens, interventions, comparators, and outcome measures [8,15,16,17,18,19,20,21,22,23,24,25,26,27,28,29,30,31,32,33]. Supplementary File S5: List of the 27 records originally classified as “reports not retrieved,” together with the documented reason for their reclassification or exclusion. Supplementary File S6: Outcome-specific RoB 2 domain judgments and supporting rationales [8,9,15,16,17,18,19,20,21,22,23,24,25,26,27,28,29,30,31,32,33]. Supplementary File S7: Additional methodological sensitivity analyses and leave-one-out plots [8,15,16,17,18,19,20,21,22,23,24,25,26,27,28,29,30,31,32,33].

## Statistical Declaration

The authors confirm that the statistical methods used in this systematic review and meta-analysis were appropriate for the study design and research objectives. The statistical analyses were reviewed by Limei Zhao. The authors take responsibility for the selection, application, and interpretation of the statistical methods used in this manuscript.
