# Supplementary material for: Non-Pharmacological Interventions for Sleep Quality and Related Symptoms in Patients with End-Stage Renal Disease: A Systematic Review and Meta-Analysis
Source: Healthcare (Basel). 2026 Sep 9;14(18):2921. doi: 10.3390/healthcare14182921 (PMC13606877; doi:10.3390/healthcare14182921)

Supplementary File S7a

Leave-One-Out Sensitivity Analysis Restricted to Studies Meeting the Prespecified Sleep-Related Population Criterion

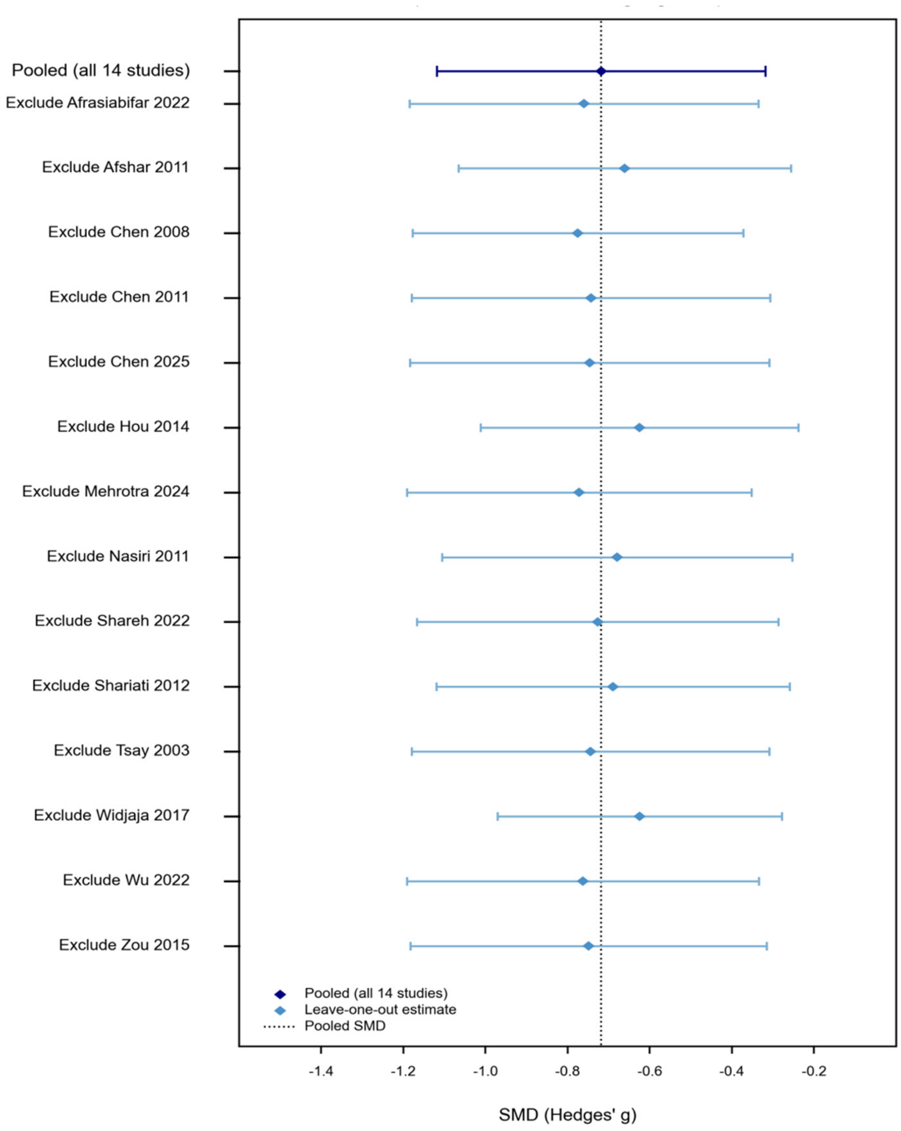

Supplementary File S7b

Leave-One-Out Sensitivity Analysis After Excluding Studies Using Active Comparators

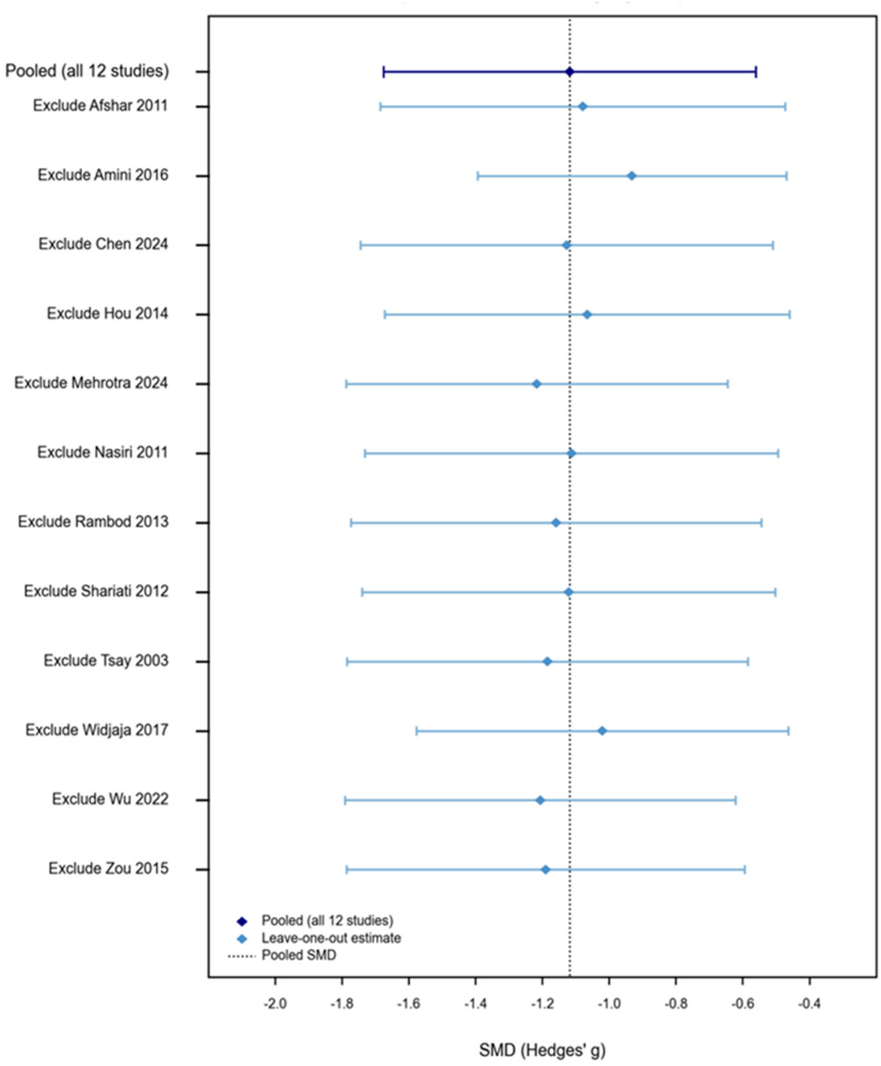

Supplementary File S7c

Leave-one-out sensitivity analysis after excluding studies at high overall risk of bias.

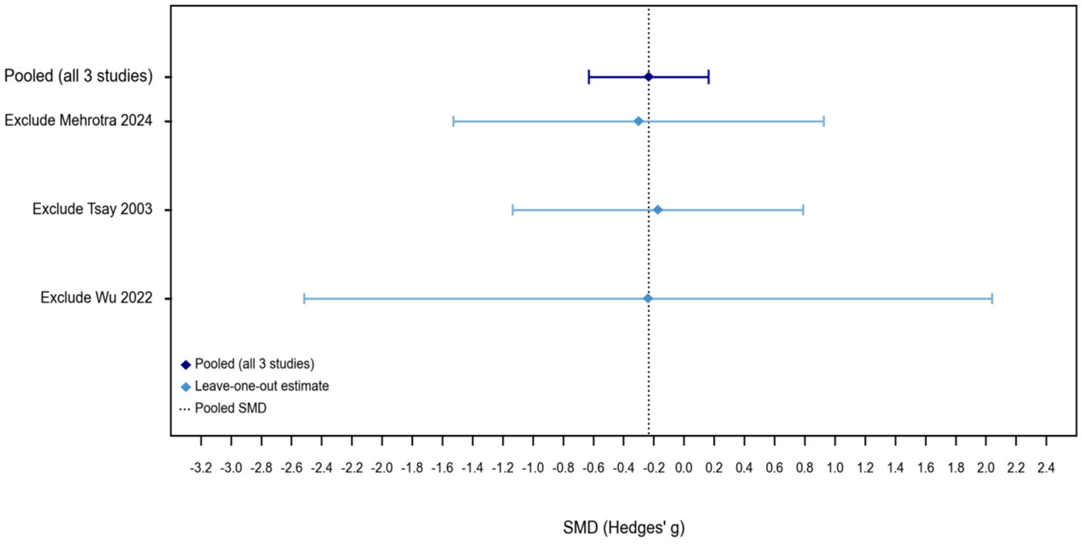

Supplementary File S7d

Leave-One-Out Sensitivity Analysis After Excluding Nakamura–Taira 2021

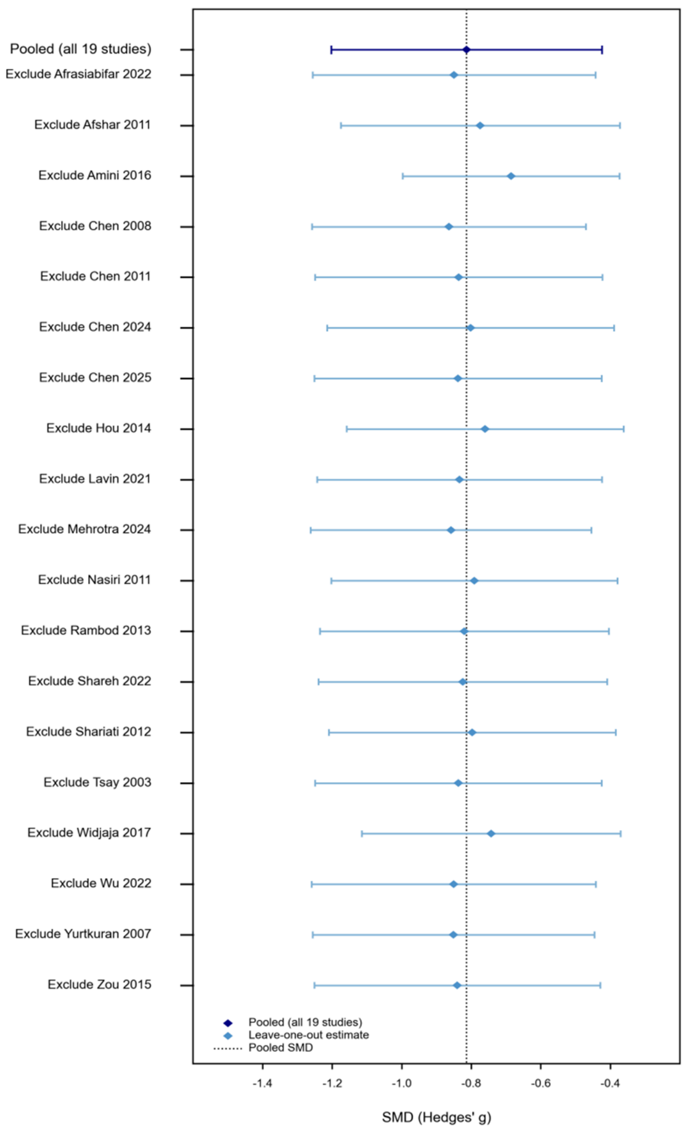

Supplement: Supplementary file 1 [file healthcare-14-02921-s001.zip › Supplementary File S7-2026.08.28.pdf]
